# Statins Attenuate *Helicobacter pylori* CagA Translocation and Reduce Incidence of Gastric Cancer: *In Vitro* and Population-Based Case-Control Studies

**DOI:** 10.1371/journal.pone.0146432

**Published:** 2016-01-05

**Authors:** Chun-Jung Lin, Wei-Chih Liao, Hwai-Jeng Lin, Yuan-Man Hsu, Cheng-Li Lin, Yu-An Chen, Chun-Lung Feng, Chih-Jung Chen, Min-Chuan Kao, Chih-Ho Lai, Chia-Hung Kao

**Affiliations:** 1 Department of Urology, University of Texas Southwestern Medical Center, Dallas, Texas, United States of America; 2 Graduate Institute of Clinical Medical Science, China Medical University, Taichung, Taiwan; 3 Department of Pulmonary and Critical Care Medicine, China Medical University Hospital, Taichung, Taiwan; 4 Division of Gastroenterology and Hepatology, Department of Internal Medicine, Taipei Medical University, Shuang-Ho Hospital, New Taipei, Taiwan; 5 Department of Biological Science and Technology, China Medical University, Taichung, Taiwan; 6 Management Office for Health Data, China Medical University Hospital, Taichung, Taiwan; 7 Graduate Institute of Basic Medical Science, School of Medicine, China Medical University, Taichung, Taiwan; 8 Department of Internal Medicine, China Medical University Hospital, Taichung, Taiwan; 9 Division of Paediatric Infectious Diseases, Department of Paediatrics, Chang Gung Children's Hospital and Chang Gung Memorial Hospital, Taoyuan, Taiwan; 10 Department of Nursing, Asia University, Taichung, Taiwan; 11 Department of Microbiology and Immunology, Graduate Institute of Biomedical Sciences, Chang Gung University, Taoyuan, Taiwan; 12 Department of Nuclear Medicine, PET Center, China Medical University Hospital, Taichung, Taiwan; University of Manitoba, CANADA

## Abstract

Gastric cancer is the second leading cause of cancer-related death worldwide. The correlation of *Helicobacter pylori* and the etiology of gastric cancer was substantially certain. Cholesterol-rich microdomains (also called lipid rafts), which provide platforms for signaling, are associated with *H*. *pylori*-induced pathogenesis leading to gastric cancer. Patients who have been prescribed statins, inhibitors of 3-hydroxy-3-methyl glutaryl coenzyme A (HMG-CoA) reductase, have exhibited a reduced risk of several types of cancer. However, no studies have addressed the effect of statins on *H*. *pylori*-associated gastric cancer from the antineoplastic perspective. In this study, we showed that treatment of gastric epithelial cells with simvastatin reduced the level of cellular cholesterol and led to attenuation of translocation and phosphorylation of *H*. *pylori* cytotoxin-associated gene A (CagA), which is recognized as a major determinant of gastric cancer development. Additionally, a nationwide case-control study based on data from the Taiwanese National Health Insurance Research Database (NHIRD) was conducted. A population-based case-control study revealed that patients who used simvastatin exhibited a significantly reduced risk of gastric cancer (adjusted odds ratio (OR) = 0.76, 95% confidence interval (CI) = 0.70–0.83). In patients exhibiting *H*. *pylori* infection who were prescribed simvastatin, the adjusted OR for gastric cancer was 0.25 (95% CI = 0.12–0.50). Our results combined an *in vitro* study with a nationwide population analysis reveal that statin use might be a feasible approach to prevent *H*. *pylori*-associated gastric cancer.

## Introduction

*Helicobacter pylori*, a Gram-negative microaerophilic spiral bacterium, colonizes the human stomach and infects over 50% of the worldwide population [1,2]. Persistent *H*. *pylori* infection is associated with several gastroenterological illnesses including gastritis, peptic ulcer, and gastric adenocarcinoma [3]. *H*. *pylori* can penetrate the mucosal layer and survive intracellularly in the gastric epithelial cells, thereby escaping host immune response or antimicrobial therapy [4,5].

Several virulence factors characterize *H*. *pylori*-induced pathogenesis [6]. Cytotoxin-associated gene A (CagA) is one of the most critical virulence factors of *H*. *pylori* [7,8]. Translocation of CagA by the *cag*-pathogenicity island (*cag*-PAI)-encoded type IV secretion system (TFSS) results in phosphorylation of the Glu-Pro-Ile-Tyr-Ala (EPIYA) motifs and induction of host cell pathogenesis, such as cell elongation (hummingbird phenotype) [9], induction of nuclear factor (NF)-κB activation, interleukin (IL)-8 secretion [10], and carcinogenesis [11].

It has been reported that *H*. *pylori* exploits cholesterol-rich microdomains (also called lipid rafts) for internalization of cells [12,13], as many pathogens do [14–16]. The major components of lipid rafts include cholesterol, phospholipids, and sphingolipids, which interact and create rigid microdomains in the cytoplasm membrane [17]. Several raft-usurping or disrupting agents such as simvastatin, methyl-β-cyclodextrin (MβCD), and filipin have been extensively employed in the investigation of the biological functions and compositions of lipid rafts [18]. Treating cells with cholesterol-usurping agents can dissociate the raft-associated proteins and lipids and render the structure nonfunctional [19]. Depletion of cholesterol has been demonstrated to attenuate CagA-induced pathogenesis, suggesting that the delivery of CagA into epithelial cells is cholesterol-dependent [20,21]. Additionally, the translocated CagA is bound to the inner leaflet of the plasma membrane through the direct binding of phosphatidylserine [13]. This indicates that *H*. *pylori* can delicately manipulate membrane cholesterol which contributes to CagA functions and pathogenesis.

According to the World Cancer Report in 2014, gastric cancer is the fifth most common cancer, and the third leading cause of cancer-related deaths, worldwide [22]. Cholesterol-rich microdomains, which provide platforms for signaling, are thought to be associated with the development of various types of cancer [23]. Additionally, cholesterol-rich rafts play a crucial role in *H*. *pylori*-induced pathogenesis and its progression to gastric cancer [24,25]. A recent population-based case-control study demonstrated that patients treated with statins that inhibit 3-hydroxy-3-methyl glutaryl coenzyme A (HMG-CoA) reductase, the rate-limiting enzyme in cholesterol biosynthesis, exhibited reduced risk of gastric cancer [26]. However, the molecular mechanism underlying statin usage attenuating the risk for *H*. *pylori*-associated gastric cancer has not been elucidated. In this study, we examined the effect of statins on gastric cancer risk in this nationwide population-based case-control study and investigated the interaction of cholesterol-lowering statins and *H*. *pylori* CagA-induced pathogenesis.

## Materials and Methods

### A. Experimental Study

#### Reagents and antibodies

CagA antibody and phosphotyrosine (4G10) antibody were purchased from Santa Cruz Biotechnology (Santa Cruz, CA, USA). Lipofectamine 2000 was purchased from Invitrogen (Carlsbad, CA, USA). Luciferase substrate and β-galactosidase expression vector were purchased from Promega (Madison, MA, USA). Simvastatin, lovastatin, RhoA inhibitor (Y27632) and all other chemicals were of the highest grade commercially available and purchased from Sigma-Aldrich (St. Louis, MO, USA).

#### Cell and bacterial culture

Human gastric epithelial cells (AGS cells, ATCC CRL 1739) were cultured in F12 (GibcoBRL, NY, USA). MKN45 cells (JCRB0254, RIKEN Cell Bank, Japan) were cultured in Dulbecco’s minimum essential medium (HyClone, Logan, UT, USA). TSGH9201 cells were cultured in RPMI1640 medium (Gibco Laboratories, Grand Island, NY, USA). Ten percent de-complemented fetal bovine serum (Hyclone) was added to all cultures. Penicillin and streptomycin (GibcoBRL) were used if necessary. Antibiotics were not added to the cell culture medium in the *H*. *pylori*-infected assay. *H*. *pylori* 26695 (ATCC 700392) were routinely cultured on Brucella blood agar plates (Becton Dickinson, Franklin Lakes, NJ, USA) containing 10% sheep blood under 5% CO_2_ and 10% O_2_ conditions at 37°C for 2–3 days.

#### Analysis of cell viability and cellular cholesterol

Gastric epithelial cells were treated with or without various concentrations of simvastatin (0, 10, 20, and 50 μM) for 1 h. Trypan blue staining was used to measure the effects of statin on cell viability [20]. After treatment with simvastatin, the cells were washed with phosphate-buffered saline (PBS) and disrupted through ultrasonication (three 10-s bursts at room temperature). The cellular cholesterol was measured using an Amplex Red cholesterol assay kit (Molecular Probes) according to the manufacturer’s instructions [21].

#### Analysis of translocated CagA and phophorylated CagA

Immunoprecipitates for analysis of *H*. *pylori* CagA translocation and phophorylation were prepared using the relevant techniques [20]. The immunoprecipitates were subjected to 6.5% SDS-PAGE and transferred onto a polyvinylidene difluoride membrane (Pall, East Hills, NY, USA) for immunoblot analysis. CagA was probed using mouse anti-CagA antibodies (Santa Cruz Biotechnology) and tyrosine-phosphorylated CagA was probed using mouse antiphosphotyrosine antibodies (4G10) (Upstate Biotechnology, Billerica, MA, USA). To ensure equal loading of each prepared sample, β-actin from whole-cell lysates was stained using goat antiactin antibodies (Santa Cruz Biotechnology). The relevant proteins were visualized using enhanced chemiluminescence reagents (GE Healthcare, Buckinghamshire, UK) and were detected by exposure to X-ray film (Kodak, Boca Raton, FL, USA).

#### Transient transfection of NF-κB reporter gene and luciferase activity assay

AGS cells were grown in 12-well plates for 20 h and transfected with a NF-κB-luc reporter plasmid using Lipofectamine 2000 (Invitrogen, Carlsbad, CA, USA) [21]. After a 24-h incubation for transfection, cells were cocultured in the presence of 25 μM simvastatin and thereafter infected with *H*. *pylori* at an multiplicities of infection (MOI) of 100 for 6 h. The cells were scraped from dishes and an equal volume of luciferase substrate (Promega) was added to the samples. The luminescence was measured using a microplate luminometer (Biotek, Winooski, VT, USA). Luciferase activity was normalized to transfection efficiency, which was determined using the β-galactosidase activity of a cotransfected β-galactosidase expression vector (Promega).

#### Measurement of IL-8

AGS cells were treated with 25 μM simvastatin and thereafter infected with *H*. *pylori* at an MOI of 100 and incubated for 16 h. The supernatants from cell cultures were collected and the levels of IL-8 were determined using a sandwich enzyme-linked immunosorbent assay (ELISA) kit (R&D Systems, Minneapolis, MN, USA) according to the manufacturer’s instructions [27].

#### Analysis of *H*. *pylori*-induced hummingbird phenotype of human gastric epithelial cells

AGS cells (1 × 10^6^ cells) were cultured in 12-well plates at 37°C for 20 h. After one wash with PBS, cells were treated with simvastatin (25 μM), then infected with *H*. *pylori* 26695 at an MOI of 100 for 6 h. Elongated cells were defined as cells that were typically elongated and had thin needle-like protrusions > 20 μm long. All samples were cultured in triplicate in 3 independent experiments. The proportion of elongated cells was determined and cells with the hummingbird phenotype were counted [20].

### B. Population-Based Case-Control Study

#### Data source

The National Health Insurance (NHI) program was implemented in 1995 and covers approximately 99% of the Taiwanese population, and has contracts with 97% of all medical providers [28]. The details of NHI program have been comprehensively described in previous reports [29,30]. The database provides diagnostic codes in the format of the International Classification of Disease, Ninth Revision, Clinical Modification (ICD-9-CM). The database encrypts the patients’ personal information for privacy protection and provides researchers with anonymous identification numbers associated with the relevant claim information, which includes the patient's sex, date of birth, registry of medical services, and medication prescriptions. Patient consent is not required for accessing the database. This study was approved by the Institutional Review Board of China Medical University (CMU-REC-101-012). Our IRB specifically waived the requirement for consent.

#### Sampled participants

In this population-based case-control study, patients aged at least 20 years, with recently diagnosed gastric cancer (ICD-9-CM code 151), were selected from the Catastrophic Illnesses Patient Database (CIPD) as the case group during the period 2005–2010. The NHI offers a catastrophic illness program that exempts patients from co-paying for the corresponding medical services, and the CIPD includes cancer patients. The index date for each case was the date of diagnosis of gastric cancer. Control subjects were identified from the Longitudinal Health Insurance Database 2000 (LHID 2000), a database containing the claims data from 1996 to 2011 for 1 million people randomly sampled from 2000 Registry of Beneficiaries of the Taiwan NHI program. For each case, one control patient, frequency-matched for sex, age (in 5-y bands), and year of gastric cancer diagnosis, was selected as the control group. Patients with any other cancer (ICD-9-CM codes 140–208) diagnosed before the index date were excluded. To explore the association between individual statins and gastric-cancer risk, the medication history of two statins commercially available before the index date, simvastatin and lovastatin, was analyzed.

#### Variables of interest

Co-morbidities were identified at least three times from principal/secondary diagnoses in the outpatient visits and/or hospitalizations. For each patient, records of co-morbidities present since the index date were obtained. Covariates included gender, age, and co-morbidities including *H*. *pylori*-infection (ICD-9-CM code 041.86), gastritis (ICD-9-CM codes 535.0, 535.1, 535.3, 535.4, 535.5), gastric diseases (ICD-9-CM codes 531–533), gastric polyp (ICD-9-CM code 211.1), gastroesophageal reflux disease (ICD-9-CM codes 530.81 and 530.11), and cirrhosis (ICD-9-CM code 571). These variables were potential confounders associated with gastric cancer.

#### Statistical analysis

The differences in sex, age (20–39 y, 40–64 y, 65–74 y, and ≥ 75 y), medication history of simvastatin and lovastatin, and co-morbidities were compared between the gastric-cancer cases and the controls by using a chi-square test. We used a *t* test for continuous variables. Univariate and multivariate unconditional logistic regression model was used to calculate the odds ratios (ORs) and 95% confidence intervals (CIs). The ORs and 95% CIs were measured to explore the risk of gastric-cancer associated with medication use and co-morbidities. The multivariate analysis was performed to adjust for possible confounders (including co-morbidities of *H*. *pylori* infection, gastric diseases, gastroesophageal reflux disease, gastric polyp, cirrhosis, and gastritis). The defined daily dose (DDD), recommended by the World Health Organization (WHO), was assumed to be the average maintenance dose per day of a drug. The annual mean DDD was calculated by diving the total cumulative DDD by the number of days in a year. Patients were classified into 3 groups according to the first quartile and second quartile of dose distribution. All analyses were conducted using SAS statistical software (Version 9.3 for Windows; SAS Institute, Inc., Cary, NC, USA). All statistical tests were performed at the 2-tailed significance level of 0.05.

## Results

### Statin Reduces Cellular Cholesterol

**Fig 1:**
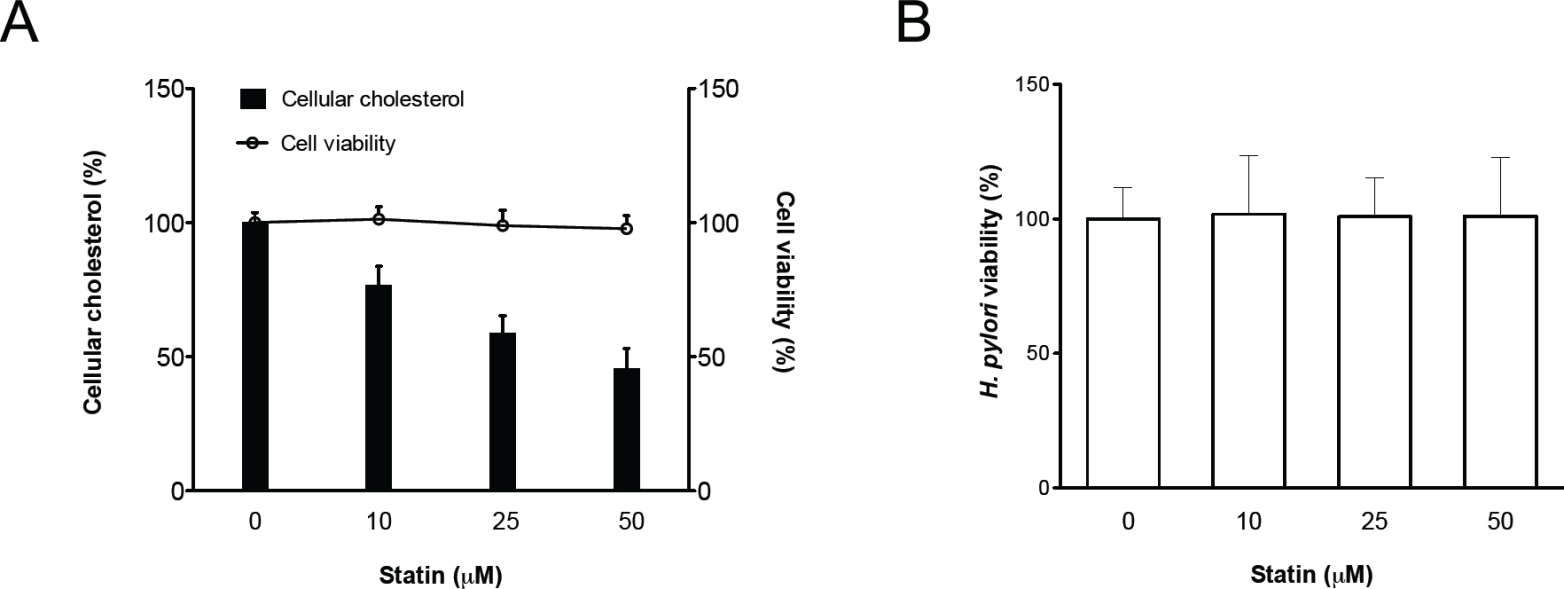
The level of cellular cholesterol in gastric epithelial cells was reduced through treatment with statins. AGS cells were treated with various concentrations of simvastatin (0–50 μM) and infected with *H*. *pylori* at an MOI of 100 for 6 h. (A) Whole cell lysates were then prepared for cholesterol level analysis (open bar). (B) Bacterial suspension was plated onto Brucella blood agar plates and incubated for 3–4 days, after which the CFUs were counted for evaluation of bacterial viability (open circle). (B) Cell viability was not influenced by treatment with simvastatin, as determined by the trypan blue exclusion assay. The data are presented as means ± standard deviations for three independent experiments. Statistical significance was evaluated using Student’s *t*-test (*, *P* < 0.05).

To analyze whether statins affect cellular cholesterol, AGS cells were pretreated with simvastatin (0–50 μM) and infected with wild-type *H*. *pylori* 26695; the levels of cellular cholesterol were then determined. As indicated in Fig 1A, simvastatin-treated cells exhibited a significantly reduced level of cellular cholesterol in a concentration-dependent manner. The viability of *H*. *pylori* and the cells was barely affected by treatment with simvastatin (Fig 1B).

### Statin Decreases *H*. *pylori* CagA Translocation and Phosphorylation

**Fig 2:**
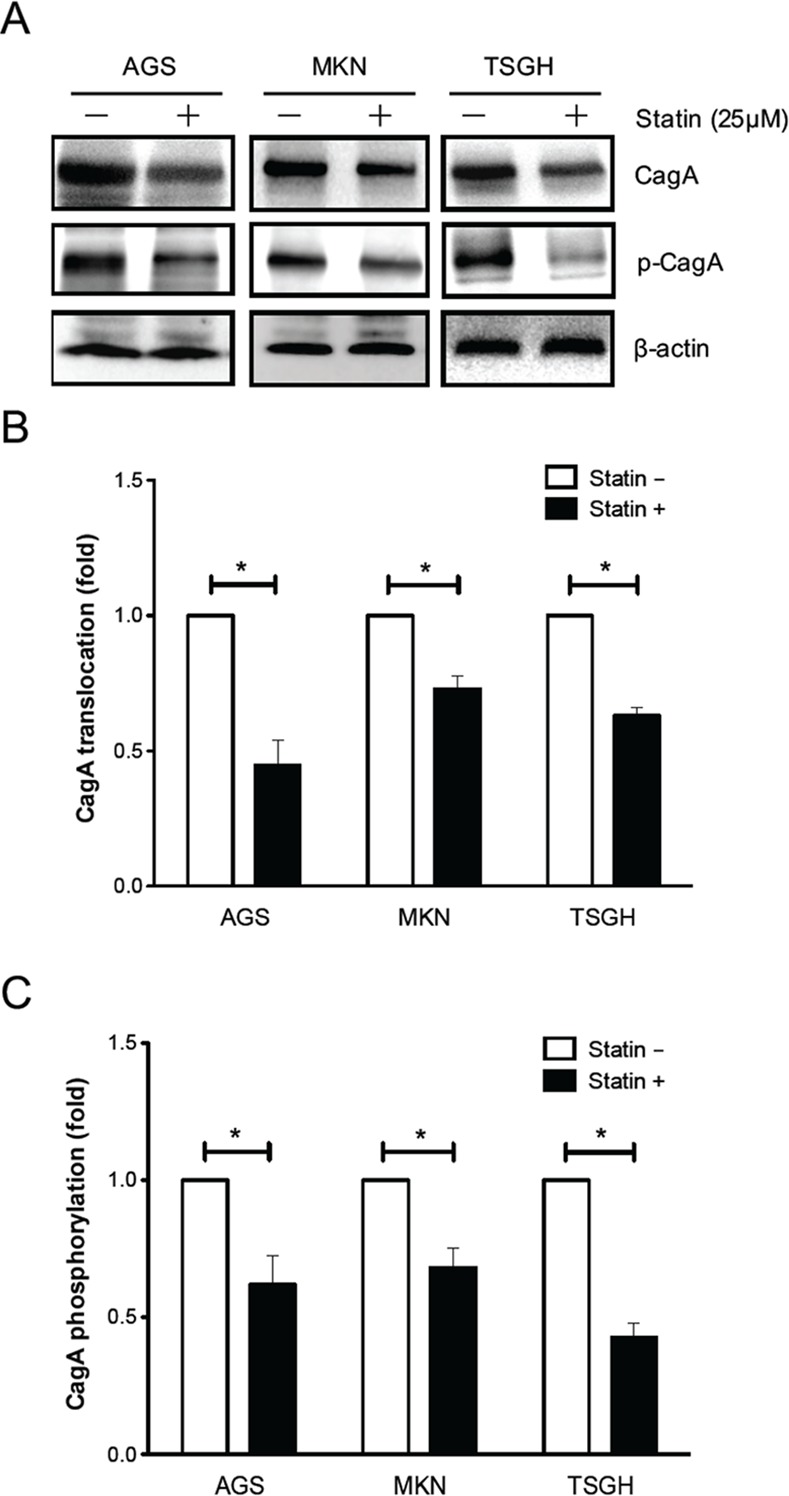
Statin decreases *H*. *pylori* CagA translocation/phosphorylation in gastric epithelial cells. Three lines of gastric epithelial cells (AGS, MKN45, and TSGH9201) were pretreated with 25 μM simvastatin, then infected with *H*. *pylori* at an MOI of 100 for 6 h. (A) Whole-cell lysates were subjected to immunoblot for analysis of CagA translocation/phosphorylation, respectively. β-actin was determined as an internal control for equal loading. The quantitative results of (B) translocated CagA and (C) phosphorylated CagA were determined using densitometric analysis and presented as means ± standard deviations for three independent experiments. Statistical analysis was performed using Student’s *t*-test. *, *P* < 0.05 compared with *H*. *pylori*-infected cells without simvastatin pretreatment.

The influence of a lower level of cellular cholesterol on translocation and phosphorylation of CagA in *H*. *pylori*-infected gastric epithelial cells was examined. As indicated in Fig 2, the levels of translocated and tyrosine-phosphorylated CagA were reduced significantly in AGS cells treated with 25 μM simvastatin. This trend was also observed in the other gastric cancer cell lines, MKN45 and TSGH9201 cells. These results indicated that the reduced levels of cellular cholesterol achieved by simvastatin attenuated CagA translocation and phosphorylation in *H*. *pylori*-infected cells.

### Statin Mitigates *H*. *pylori*-Induced Pathogenesis

**Fig 3:**
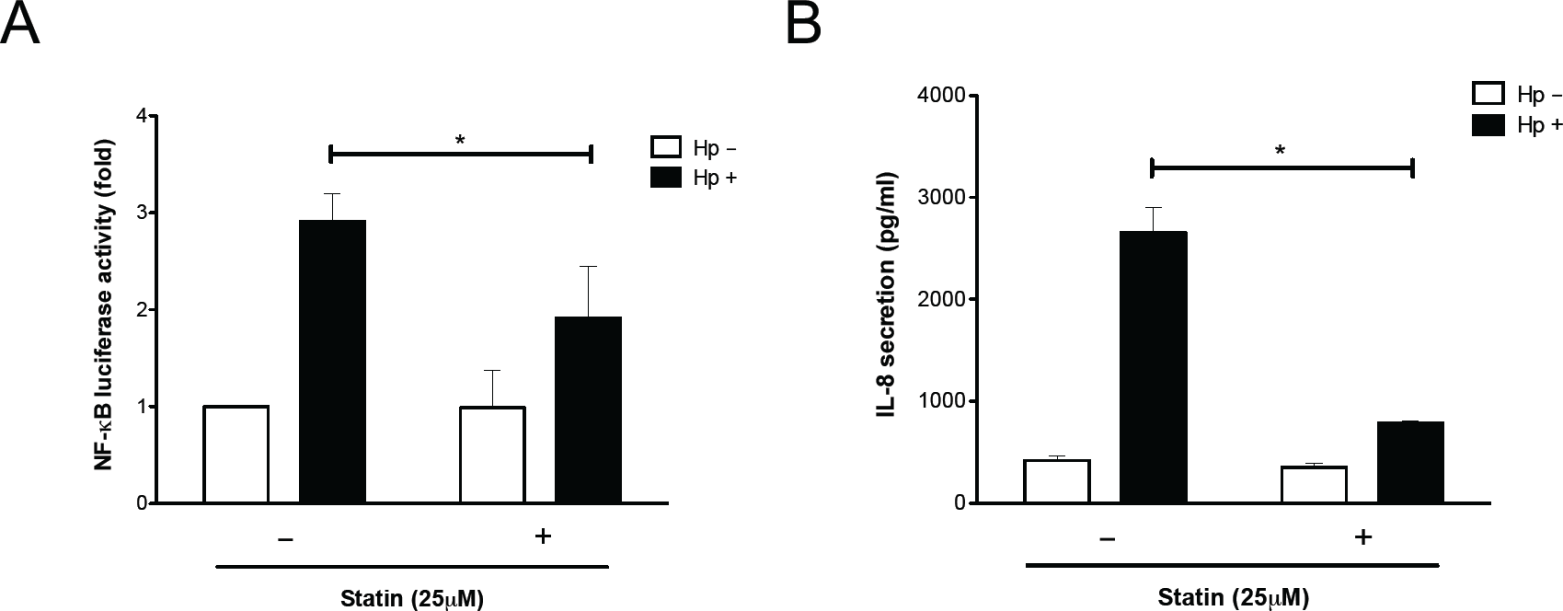
Statin attenuates *H*. *pylori* CagA-induced pathogenesis. (A) AGS cells were transfected with κB-Luc vector and incubated for 24 h. The cells were then treated with 25 μM simvastatin, and infected with *H*. *pylori* at an MOI of 100 for 6 h. The cells were then prepared for luciferase activity assays. (B) AGS cells were pretreated with simvastatin (25 μM) prior to infection with *H*. *pylori* at an MOI of 100 for 16 h. The concentration of IL-8 in the culture supernatant was analyzed using the ELISA method. Results are expressed as means ± standard deviations. *, *P* < 0.05 was considered statistically significant.

**Fig 4:**
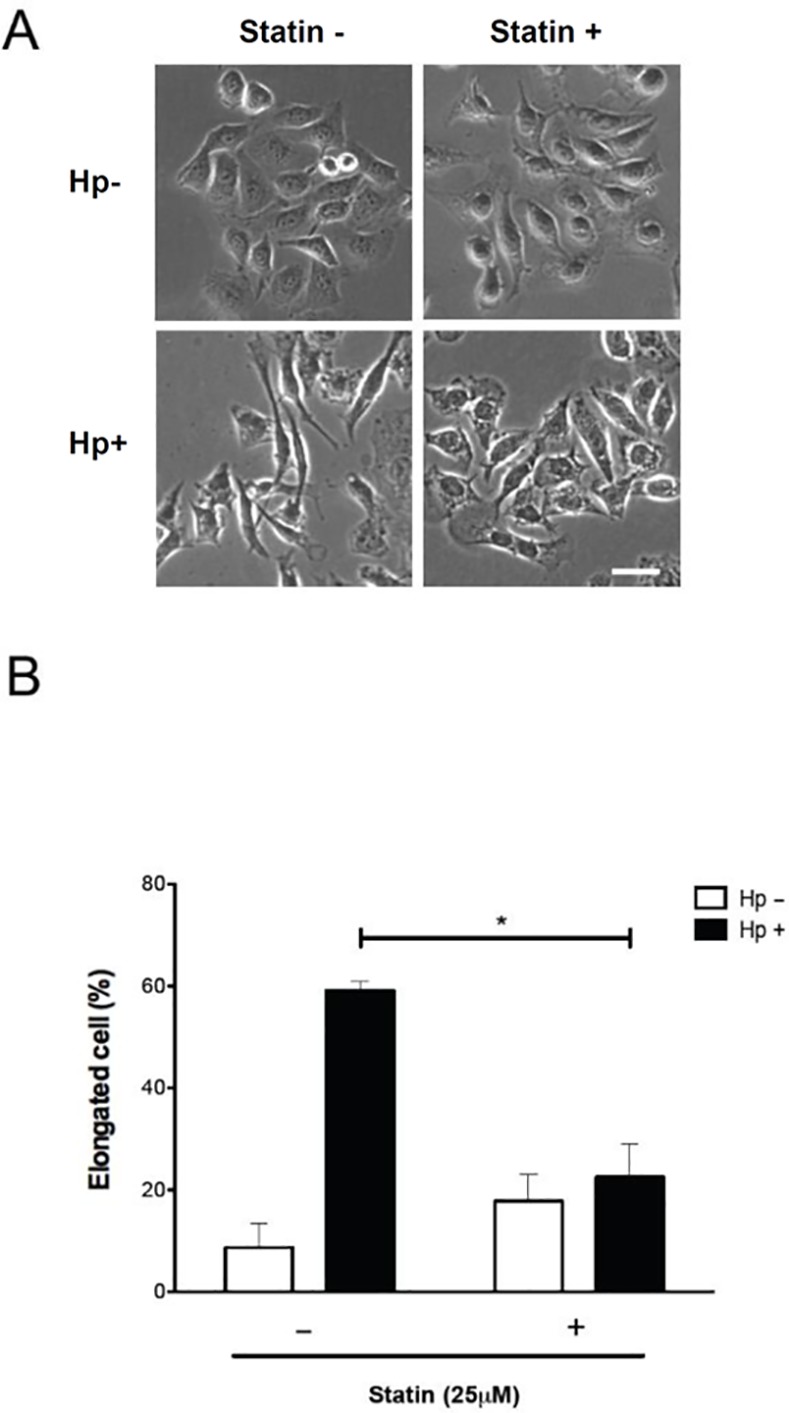
Statin reduces *H*. *pylori*-induced elongation of gastric epithelial cells. (A) AGS cells were pretreated with simvastatin (25 μM) and infected with *H*. *pylori* at an MOI of 100 for 6 h. (B) The proportion of cells with the elongated (hummingbird) phenotype was evaluated as described in the materials and methods section. The quantitative results represent the means and standard deviations for three independent experiments. *, *P* < 0.05, compared with *H*. *pylori*-infected cells without simvastatin pretreatment. Scale bar, 10 μm.

The translocated/phosphorylated CagA in gastric epithelial cells is associated with activation of NF-κB and production of IL-8, followed by induction of hummingbird phenotype formation [10]. To investigate the mechanism responsible for statin-mediated inhibition of *H*. *pylori* CagA functions, NF-κB luciferase activity and IL-8 production were analyzed. The results revealed that treatment of cells with 25 μM simvastatin and subsequent infection with *H*. *pylori* reduced the levels of NF-κB promoter activity and secretion of IL-8 (Fig 3). We then examined whether simvastatin, in addition to inhibiting translocation and phosphorylation of CagA, also specifically attenuates CagA-induced responses by evaluating the hummingbird phenotype of cells. AGS cells were pretreated with or without simvastatin (25 μM) and subsequently infected with *H*. *pylori*. Approximately 60% of cells represented the hummingbird phenotype when compared with the uninfected cells (Fig 4). In cells pretreated with 25 μM simvastatin, the proportion of *H*. *pylori*-induced cell elongation was substantially reduced. Because *H*. *pylori* CagA can stimulate RhoA-dependent activation of NF-κB [31,32], we further investigated the role of the RhoA inhibitor in our findings. As shown in S1 Fig, *H*. *pylori*-induced NF-κB promoter activity was markedly reduced in cells treated with either simvastatin or RhoA inhibitor compared to that in the untreated group. In parallel, treatment with simvastatin and RhoA inhibitor also significantly attenuated *H*. *pylori* CagA-induced hummingbird phenotype (S2 Fig). These results are in agreement with those of previous reports where RhoA was shown to mediate the inhibitory effect of statin [33,34], indicating that statin not only reduced cellular cholesterol but also inhibited cholesterol pathway intermediates metabolites. Taken together, our findings indicated that simvastatin reduced cellular cholesterol and inhibited the cholesterol pathway intermediates that mitigated the geranylgeranylated RhoA-dependent activation of NF-κB, leading to attenuation of CagA translocation/phosphorylation levels and reduction in the hummingbird phenotype of *H*. *pylori*-infected cells.

### Demographic Analyses

A total of 19728 patients with recently diagnosed gastric cancer were identified from 2005 to 2010 and 19727 patients comprised the control group. Among the enrolled patients, 63.0% were men and 61.0% were aged 65 years or older (Table 1). The mean age (± SD) was 67.7 ± 14.2 years for patients diagnosed with gastric cancer and 67.3 ± 14.4 years for the control patients. The proportion of patients with medication history of simvastatin and lovastatin was lower in the gastric-cancer group than in the control group. Patients diagnosed with gastric cancer exhibited a significantly higher prevalence of co-morbidities than did the controls including *H*. *pylori* infection (3.41% vs 0.38%), gastric diseases (75.3% vs 40.4%), gastroesophageal reflux disease (14.6% vs 5.00%), gastric polyp (2.65% vs 0.37%), cirrhosis (31.9% vs 26.6%), and gastritis (54.1% vs 40.6%).

**Table 1:** Baseline characteristics between gastric cancer group and non-gastric cancer group.

	Gastric cancer				
	No		Yes		
	n = 19727		n = 19728		
	n	%	n	%	*P* value^†^
Gender					0.99
Women	7305	37.0	7306	37.0	
Men	12422	63.0	12422	63.0	
Age group (year)					0.99
20–39	673	3.41	672	3.41	
40–64	7013	35.6	7014	35.6	
65–74	4641	23.5	4641	23.5	
≥75	7400	37.5	7401	37.5	
Mean^†^	67.3	14.4	67.7	14.2	0.0009
Medications					
Simvastatin	1458	7.39	1305	6.61	0.003
Lovastatin	1472	7.46	1428	7.24	0.40
Baseline co-morbidities					
*H*. *pylori*-infection	74	0.38	673	3.41	<0.0001
Gastric diseases	7970	40.4	14849	75.3	<0.0001
Gastroesophageal reflux disease	987	5.00	2872	14.6	<0.0001
Gastric polyp	73	0.37	523	2.65	<0.0001
Cirrhosis	5240	26.6	6291	31.9	<0.0001
Gastritis	8011	40.6	10662	54.1	<0.0001

^†^Chi-square test and Student’s *t*-test comparing subjects with and without gastric cancer. Data are presented as the number of subjects in each group with percentages.

### Statin Use Reduces the Risk of Gastric Cancer

Patients who used simvastatin exhibited a significantly reduced risk of gastric cancer (adjusted OR = 0.76, 95% CI = 0.70–0.83) (Table 2). A reduced risk of gastric cancer in patients prescribed lovastatin, compared with those who did not use lovastatin, was also observed (adjusted OR = 0.79, 95% CI = 0.72–0.87). In the multivariate analysis, *H*. *pylori* infection (adjusted OR = 5.09, 95% CI = 3.98–6.51), gastric diseases (adjusted OR = 4.00, 95% CI = 3.82–4.19), gastroesophageal reflux disease (adjusted OR = 2.13, 95% CI = 1.97–2.31), gastric polyp (adjusted OR = 5.14, 95% CI = 3.98–6.62), and gastritis (adjusted OR = 1.15, 95% CI = 1.10–1.20) were associated with increased odds of gastric cancer. We performed an analysis of the dose-related response, using nonusers of statins as the reference group (Table 3). Compared with nonusers of statins, patients receiving simvastatin treatment exhibited a significant reduction in the risk of gastric cancer in all dose groups (adjusted OR = 0.70, 95% CI = 0.59–0.83 for < 5 DDD; adjusted OR = 0.76, 95% CI = 0.65–0.88 for 5–25 DDD; and adjusted OR = 0.79, 95% CI = 0.70–0.88 for ≥ 25 DDD). Lovastatin was also associated with a significant reduction in the risk of gastric cancer in all dose groups (adjusted OR = 0.84, 95% CI = 0.72–0.98 for < 5 DDD; adjusted OR = 0.78, 95% CI = 0.67–0.91 for 5–15 DDD; and adjusted OR = 0.80, 95% CI = 0.71–0.90 for ≥ 25 DDD). We then determined whether the association between statin use and the risk of gastric cancer differs in patients diagnosed with *H*. *pylori* infection (Table 4). The results indicated that in patients diagnosed with *H*. *pylori* infection, the adjusted OR for gastric cancer in patients prescribed simvastatin was 0.25 (95% CI = 0.12–0.50), compared with those not prescribed simvastatin. The same trend was also observed in patients treated with lovastatin, but the reduction in risk was nonsignificant. We further analyzed whether the use of statin was associated with the occurrence of different types of gastric cancers. As shown in Table 5, patients who used simvastatin exhibited a significantly reduced risk of the proximal type, distal type, and other types of gastric cancers. In addition, patients who were prescribed lovastatin showed a lower risk of other types of gastric cancer compared to those who did not use lovastatin.

**Table 2:** Odds ratios and 95% confidence intervals of gastric cancer associated with simvastatin, lovastatin and covariates.

	Crude		Adjusted^†^	
Variable	OR	(95% CI)	OR	(95% CI)
Medications				
Simvastatin	0.89	(0.82, 0.96)**	0.76	(0.70, 0.83)***
Lovastatin	0.97	(0.90, 1.04)	0.79	(0.72, 0.86)***
Baseline co-morbidities				
*H*. *pylori*-infection	9.38	(7.37, 11.9)***	5.09	(3.98, 6.51)***
Gastric diseases	4.49	(4.30, 4.69)***	4.00	(3.82, 4.19)***
Gastroesophageal reflux disease	3.24	(3.00, 3.49)***	2.13	(1.97, 2.31)***
Gastric polyp	7.32	(5.73, 9.36)***	5.14	(3.98, 6.62)***
Cirrhosis	1.29	(1.24, 1.35)***	0.95	(0.90, 1.00)
Gastritis	1.72	(1.65, 1.79)***	1.15	(1.10, 1.20)***

^†^Adjusted for *H*. *pylori*-infection, gastric diseases, gastroesophageal reflux disease, gastric polyp, and gastritis.

**, *P* < 0.01

***, *P* < 0.001.

Abbreviations: CI, confidence intervals; OR, odds ratios.

**Table 3:** Odds ratio and 95% confidence intervals of gastric cancer associated with annual mean DDD use of individual statins.

	Case number/control number	Crude odds ratio	(95% CI)	Adjusted odds ratio^†^	(95% CI)
Non-use of statins	17402/17291	1.00	(Reference)	1.00	(Reference)
Simvastatin					
<5 DDD	278/318	0.86	(0.74, 1.02)	0.70	(0.59, 0.83)***
5–25 DDD	399/440	0.90	(0.79, 1.03)	0.76	(0.65, 0.88)***
≥ 25 DDD	628/700	0.89	(0.80, 0.99)*	0.79	(0.70, 0.88)***
*P* for trend					< 0.001
Lovastatin					
<5 DDD	410/408	1.00	(0.87, 1.15)	0.84	(0.72, 0.98)*
5–15 DDD	392/389	1.00	(0.87, 1.15)	0.78	(0.67, 0.91)**
≥ 15 DDD	626/675	0.92	(0.83, 1.03)	0.80	(0.71, 0.90)***
*P* for trend					< 0.001

^†^Adjusted for *H*. *pylori* infection, gastric diseases, gastroesophageal reflux disease, gastric polyp, and gastritis.

*, *P* < 0.05

**, *P* < 0.01

***, *P* < 0.001.

Abbreviations: CI, confidence intervals; DDD, defined daily dose.

**Table 4:** Joint effect between statin treatment and co-morbidities on risk of gastric cancer.

Variables		Event (n)	Event (n)	Adjusted odds ratio^†^ (95% CI)
Simvastatin treatment	*H*. *pylori-*infection			
No	Yes	698	638	1 (Reference)
Yes	Yes	49	35	0.25 (0.12, 0.50)***
Lovastatin treatment	*H*. *pylori-*infection			
No	Yes	690	623	1 (Reference)
Yes	Yes	57	50	0.85 (0.36, 2.00)

^†^Adjusted for gastric diseases, gastroesophageal reflux disease, gastric polyp, and gastritis.

***, *P* < 0.001.

Abbreviation: CI, confidence intervals.

**Table 5:** Odds ratios and 95% confidence intervals of different types of gastric cancer associated with statin use.

Variables^†^	Crude OR (95% CI)	Adjusted OR^‡^ (95% CI)
Non-use of statins	1.00	1.00
Proximal stomach (N = 7803)		
Simvastatin	0.95 (0.86, 1.05)	0.82 (0.73, 0.91)***
Lovastatin	1.13 (1.02, 1.24)*	0.94 (0.85, 1.05)
Distal stomach (N = 4539)		
Simvastatin	0.81 (0.71–0.93)**	0.65 (0.57, 0.75)***
Lovastatin	1.09 (0.97, 1.23)	0.88 (0.78, 1.00)
Others (N = 30925)		
Simvastatin	0.99 (0.92, 1.06)	0.83 (0.77, 0.89)***
Lovastatin	0.96 (0.89, 1.02)	0.78 (0.72, 0.84)***

^†^Proximal stomach: ICD-9-CM 151.0, 151.3, 151.5, 151.6; Distal stomach: ICD-9-CM 151.1, 151.2; Others: ICD-9-CM 151.8, 151.9.

^‡^Adjusted for *H*. *pylori* infection, gastric diseases, gastroesophageal reflux disease, gastric polyp, and gastritis.

*, *P* < 0.05

**, *P* < 0.01

***, *P* < 0.001.

Abbreviation: OR, odds ratios; CI, confidence intervals.

## Discussion

The inhibitors of HMG-CoA reductase, commonly known as statins, are widely prescribed for lowering serum cholesterol. Statins are associated with multiple protective functions including reducing the risk of hepatocellular carcinoma [35], enhancing chemosensitivity in colorectal cancer [36], and reducing the risk of death in patients with prostate cancer [37]. The results of this study consistently indicated that the use of statins may reduce gastric cancer risk. The results of previous studies implying an inverse association between statin use and the risk of gastric cancer have been nonsignificant [38,39]. In addition, these reports were based on small sample sizes and did not adjust for potential confounders. In contrast, the totality of epidemiologic evidence synthesized in recent meta-analyses on this topic support a statistically significant inverse association between statin use and gastric cancer risk [40,41]. We used a nationwide database and extended the period from 2005 to 2010 for further analysis. A total of 19728 patients with gastric cancer and 19727 patients without gastric cancer were enrolled in this study. Additionally, the multivariate analysis was adjusted for several possible confounders, including co-morbidities of *H*. *pylori* infection, gastric diseases, gastroesophageal reflux disease, and gastritis. Therefore, the results of the present study were highly reliable and consistent with the findings of Chiu *et al*, who reported the association of statin use with reduced gastric cancer risk by conducting a population-based case–control study [26].

The mechanisms underlying the immunoregulatory effects of statins have been studied by other researchers. Exposure of murine macrophages to statins attenuated lipopolysaccharide (LPS)-induced proinflammatory cytokine production [42]. Moreover, LPS-challenge serum levels of IL-1β and TNF-α, and monocyte-activating Toll-like receptor 2 (TLR2) and TLR4 were decreased in humans treated with statins [43]. Treatment of *Staphylococcus aureus* with high concentrations of statins resulted in considerable antimicrobial activity [44]. However, in this study, bacterial viability did not influence the treatment of *H*. *pylori* with 0–50 μM statins (Fig 1A). CagA is the major determining factor for NF-κB activation followed by IL-8 secretion, and is critical for *H*. *pylori*-induced gastric inflammation [10]. Previous studies have indicated that depletion of cholesterol by lipid raft-disrupting/usurping agents significantly attenuates CagA-mediated pathogenesis consistently [20,21]. Therefore, it is possible that the anti-*H*. *pylori*–induced-inflammation action of statins was mediated by a depletion of cholesterol which might inhibit CagA-functions in host cells rather than *H*. *pylori* growth.

Statins are known to reduce the risk of severe bacterial infections, including *Chlamydia pneumonia* [45], *Clostridium difficile* [46], *Streptococcus pneumonia* [47], and *Staphylococcus aureus* [48]. The immunomodulatory properties of statins only partly explain the potential antiinfection mechanism. This paper indicates that statins reduced NF-κB promoter activity and IL-8 production (Fig 3). These results are supported by previous studies indicating that statins might influence transcriptional factors, AP-1 and NF-κB, thereby reducing the secretion of proinflammatory cytokines [49]. Additionally, statin-treatment was observed to inhibit *H*. *pylori*-induced gastritis in mice [50]. Long-term statin therapy reduces the severity of chronic gastritis, even in the presence of *H*. *pylori* infection [51]. Statins can be used as adjuvants to improve the *H*. *pylori* eradication rates of standard antibiotics [52]. Although these studies have explained the pleiotropic functions exerted by statins, the exact molecular mechanism(s) of eradication of *H*. *pylori* and reduced *H*. *pylori-*associated diseases required further investigation.

The incidence and mortality rate of gastric cancer remain high worldwide. The association of statins with a reduced risk of gastric cancer has been reported by epidemiological meta-analysis studies [26,41,53]. There are several mechanisms of antineoplastic actions of statins, including upregulation of p21 and p27 [54], and inhibition of MYC phosphorylation/activation [55], which might attenuate mitosis and lead to apoptosis. Although the correlation of *H*. *pylori* etiology and gastric cancer is known, the exact mechanism of statins’ influence on *H*. *pylori*-associated gastric cancer remains unclear. *H*. *pylori* CagA is a bacterial oncoprotein that can contribute to the development of *H*. *pylori*-associated neoplasms in mammals [11]. Furthermore, it is well documented that the delivery of CagA into cells by bacterial TFSS requires membrane cholesterol [5,13,20]. Therefore, it was suggested that inhibition of cholesterol reduces CagA delivery, which might lead to the failure of *H*. *pylori*-induced carcinogenesis. The results of this study revealed that treatment of cells with statins reduces cellular cholesterol and inhibits *H*. *pylori* CagA actions, which might exert a protective effect against gastric cancer.

Studies on the various effects of statins are contradictory. Some studies have indicated that statins do not reduce the risk of gastric cancer [38,56]. In a study on *H*. *pylori*-infected rodent models, pitavastatin was ineffective in inhibiting gastric carcinogenesis [57]. This might have been due to the use of distinct types of statin for analysis because pitavastatin was not commercially available in Taiwan [53].

In this study, we found that simvastatin reduced the risks of both distal and proximal gastric cancer (Table 5). The data indicated that simvastatin might influence not only *H*. *pylori*-associated distal gastric cancer, but might also affect *H*. *pylori*-independent proximal gastric cancer. We further showed simvastatin use resulted in much lower odds ratios for distal gastric cancer than for proximal cancer (OR = 0.65 vs. 0.82). Our results demonstrated that the inhibition of cancer development by simvastatin was more pronounced in distal than in proximal gastric cancer, suggesting that simvastatin may affect *H*. *pylori*-induced pathogenesis. Although *H*. *pylori* infection is associated with distal gastric cancer, it is not the only risk factor for development of gastric cancer. The main risk factors for distal gastric cancer include *H*. *pylori* infection and diet, while gastroesophageal reflux disease and obesity play important roles in proximal gastric cancer [58]. Previous studies reported that statin use reduces the risk of Barrett's esophagus, which is a complication of gastroesophageal reflux disease [59] and associated with obesity [60]. Moreover, the mevalonate pathway has recently been reported as truly oncogenic [61], indicating that mevalonate pathway inhibitors, such as statins, are bona fide anticancer drugs [62]. These lines of evidence may explain why statin use could reduce the incidence rate of proximal gastric cancer independent of *H*. *pylori* infection. Long-term follow-up studies or randomized controlled trials are necessary to elucidate the role of statins in the prevention of different types of gastric cancer.

Some limitations might have been present in those studies, including potential confounders, concomitant use of other chemopreventive drugs, short enrolment periods, and small sample sizes. In particular, individuals of higher socioeconomic classes are more likely to be prescribed statins. A social gradient in statin use may reflect social inequalities in health care use or health care quality, as well as systematic differences in factors such as smoking, nutrition, physical activity, and obesity [63]. In addition, most of the tests performed to confirm *H*. *pylori* infection (such as serology, biopsy, urea breath test or a combination of both) are not covered by the insurance system. The data regarding *H*. *pylori* infection was under-estimated using the NHIRD. Therefore, large and long-term clinical studies on statin usage are required to further clarify statins’ mechanism of inhibiting *H*. *pylori*-associated gastric cancer.

In conclusion, we determined that *H*. *pylori* infection is associated with a high risk of gastric cancer, which is attenuated by the prescription of statins. It was also demonstrated that statins inhibit *H*. *pylori*-induced pathogenesis by preventing CagA actions in host cells. Therefore, statins, which are cholesterol-lowering agents and proven to be extremely safe, can be used as chemopreventive drugs for protecting against *H*. *pylori*-gastric cancer.

## Supporting Information

S1 Fig Statin and RhoA inhibitor mitigate *H*. *pylori* CagA-induced NF-κB activation. AGS cells were transfected with κB-Luc vector and incubated for 24 h. The cells were then treated with 25 μM simvastatin or 10μM RhoA inhibitor (Y27632), followed by infection with *H*. *pylori* at an MOI of 100 for 6 h. The cells were then prepared for luciferase activity assays. Results are expressed as means ± standard deviations. *, *P* < 0.05 was considered statistically significant.
(TIF)

S2 Fig Statin and RhoA inhibitor reduces *H*. *pylori*-induced elongation of gastric epithelial cells. (A) AGS cells were pretreated with simvastatin (25 μM) or RhoA inhibitor (Y27632, 10μM, and then infected with *H*. *pylori* at an MOI of 100 for 6 h. (B) The proportion of cells with the elongated (hummingbird) phenotype was evaluated as described in the materials and methods section. The quantitative results represent the means and standard deviations for three independent experiments. *, *P* < 0.05, compared with *H*. *pylori*-infected cells without simvastatin pretreatment. Scale bar, 10 μm.
(TIF)
