# Supplementary figures and images for: Statins Attenuate Helicobacter pylori CagA Translocation and Reduce Incidence of Gastric Cancer: In Vitro and Population-Based Case-Control Studies
Source: PLoS One. 2016 Jan 5;11(1):e0146432. doi: 10.1371/journal.pone.0146432 (PMC4701455; doi:10.1371/journal.pone.0146432)

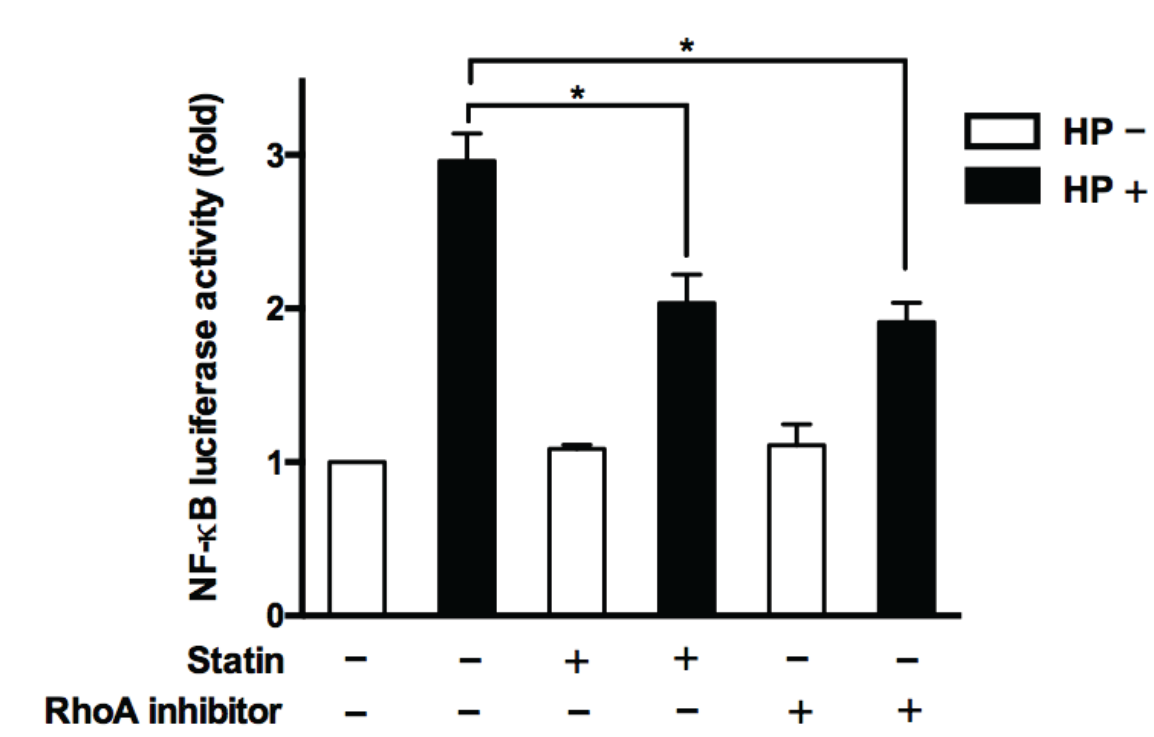

Supplement: S1 Fig — AGS cells were transfected with κB-Luc vector and incubated for 24 h. The cells were then treated with 25 μM simvastatin or 10μM RhoA inhibitor (Y27632), followed by infection with H. pylori at an MOI of 100 for 6 h. The cells were then prepared for luciferase activity assays. Results are expressed as means ± standard deviations. *, P < 0.05 was considered statistically significant. (TIF) [file pone.0146432.s001.tif]

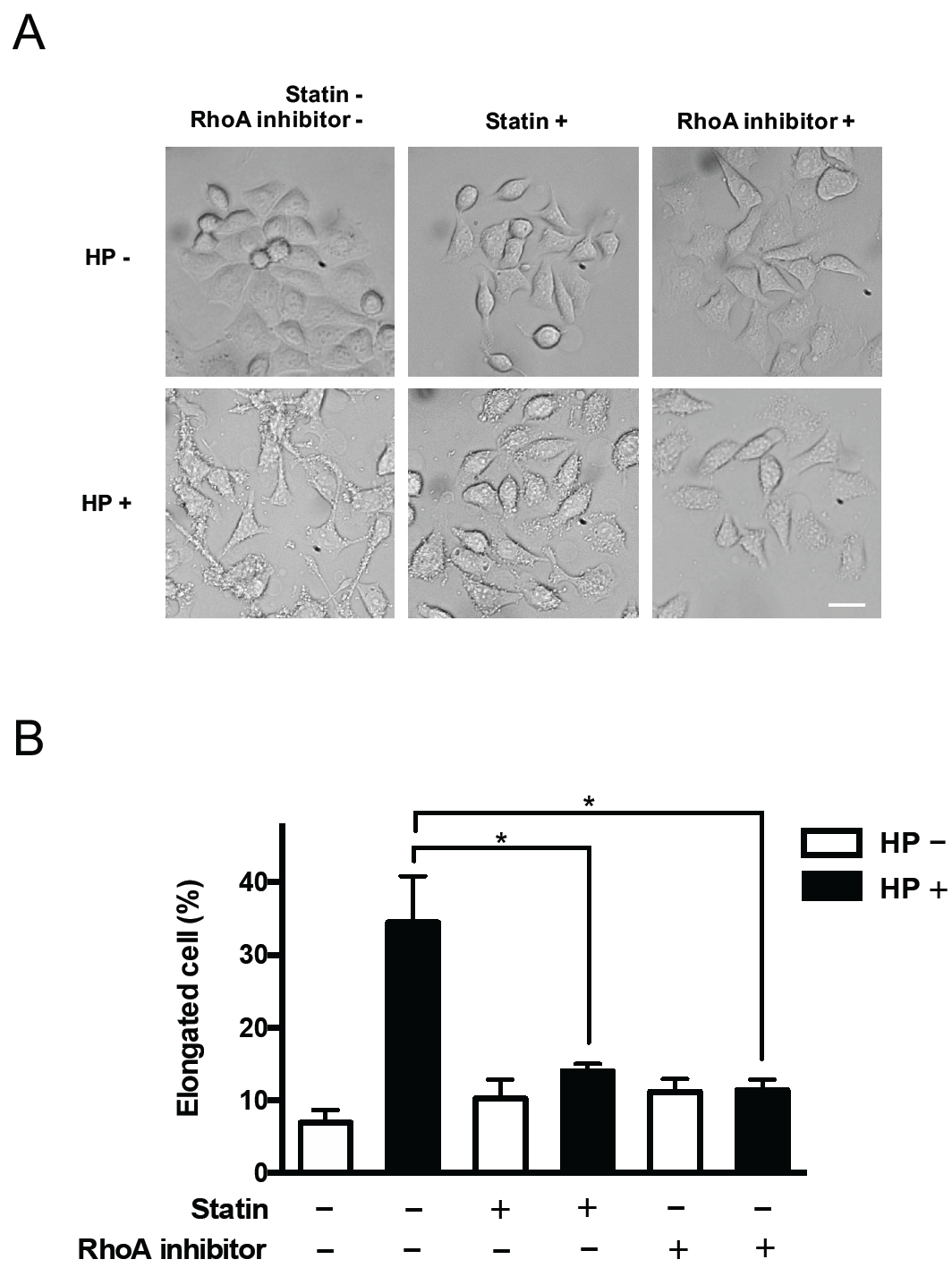

Supplement: S2 Fig — (A) AGS cells were pretreated with simvastatin (25 μM) or RhoA inhibitor (Y27632, 10μM, and then infected with H. pylori at an MOI of 100 for 6 h. (B) The proportion of cells with the elongated (hummingbird) phenotype was evaluated as described in the materials and methods section. The quantitative results represent the means and standard deviations for three independent experiments. *, P < 0.05, compared with H. pylori-infected cells without simvastatin pretreatment. Scale bar, 10 μm. (TIF) [file pone.0146432.s002.tif]
